# Lessons learned: establishing a CLIA-equivalent laboratory for targeted mass spectrometry assays – navigating the transition from research to clinical practice

**DOI:** 10.1186/s12014-024-09455-y

**Published:** 2024-02-22

**Authors:** Chia-Li Han, Chi-Ting Lai, Aaron James Reyes, Hao-Chin Yang, Jin-Ying Lu, Shyang-Rong Shih, Kuen-Yuan Chen, Andrew N. Hoofnagle, Sung-Liang Yu, William Bocik, Tara Hiltke, Huan-Chi Chiu, Ching-Yi Wan, Henry Rodriguez, Victoria Zhang, Yu-Ju Chen

**Affiliations:** 1 Master Program in Clinical Genomics and Proteomics, College of Pharmacy, Taipei Medical University, Taipei, Taiwan; 2 Institute of Chemistry, Academia Sinica, Taipei, Taiwan; 3 Division of Endocrinology and Metabolism, Department of Internal Medicine, National Taiwan University Hospital, Taipei, Taiwan; 4 Department of Surgery, National Taiwan University Hospital, Taipei, Taiwan; 5 Department of Laboratory Medicine and Pathology, University of Washington, Seattle, WA USA; 6 Department of Clinical Laboratory Sciences and Medical Biotechnology, National Taiwan University College of Medicine, Taipei, Taiwan; 7 Centers for Genomics and Precision Medicine, National Taiwan University, Taipei, Taiwan; 8 Cancer Research Technology Program, Proteome Characterization Laboratory, Frederick National Laboratory for Cancer Research, Frederick, MD USA; 9 Office of Cancer Clinical Proteomics Research, National Cancer Institute, Rockville, MD USA; 10 Department of Pathology and Laboratory Medicine, University of Rochester Medical Center, Rochester, NY USA; 11 Department of Chemistry, National Taiwan University, Taipei, Taiwan

**Keywords:** Targeted mass spectrometry, Thyroglobulin assay, Clinical laboratory improvement amendments (CLIA), Immuno-multiple reaction monitoring (iMRM) mass spectrometry

## Abstract

Mass spectrometry (MS) assays offer exceptional capabilities in high multiplexity, specificity, and throughput. As proteomics technologies continue advancements to identify new disease biomarkers, transition of these innovations from research settings to clinical applications becomes imperative. To meet the rigorous regulatory standards of clinical laboratories, development of a clinical protein MS assay necessitates adherence to stringent criteria. To illustrate the process, this project focused on using thyroglobulin (Tg) as a biomarker and an immuno-multiple reaction monitoring (iMRM) MS-based assay as a model for establishing a Clinical Laboratory Improvement Amendments (CLIA) compliant laboratory within the Centers of Genomic and Precision Medicine, National Taiwan University. The chosen example also illustrates the clinical utility of MS assays to complement conventional immunoassay-based methods, particularly in cases where the presence of autoantibodies in 10–30% of patients hinders accuracy. The laboratory design entails a comprehensive coordination in spatial layout, workflow organization, equipment selection, ventilation systems, plumbing, electrical infrastructure, documentation procedures, and communication protocols. Practical aspects of the transformation process, including preparing laboratory facilities, testing environments, instrument validation, assay development and validation, quality management, sample testing, and personnel competency, are discussed. Finally, concordant results in proficiency testing demonstrate the harmonization with the University of Washington Medical Center and the quality assurance of the CLIA-equivalent Tg-iMRM MS assay established in Taiwan. The realization of this model protein MS assay in Taiwan highlights the feasibility of international joint development and provides a detailed reference map to expedite the implementation of more MS-based protein assays in clinical laboratories for patient care.

## Introduction

It has been estimated that more than 70% of clinical decisions are based on laboratory tests [1]. In the case of cancer, one of the most predominant diseases and the second leading cause of death globally [2], early detection and accurate prognosis can significantly reduce cancer-related mortality and guide the best treatment decisions, creating better clinical and economic benefits for patients. In the current clinical setting, immunoassay methodologies using blood specimens are the most popular gold-standard protocol, yet the number of available molecular diagnosis assays is still limited. On the technical aspect, moreover, it suffers from the inherent limitations in quality of antibody and presence of antibody in patients. For example, measurement of elevated thyroglobulin (Tg) protein concentration in blood using immunometric assay is an integral part of follow up to monitor residual or recurrence for patients with differentiated thyroid cancer (DTC) [3]. However, the anti-thyroglobulin autoantibodies (TgAbs) are present in 20–30% of patients [4], which interfere with the Tg immunometric assays and potentially cause their false low results in underestimated Tg concentrations. Thus, accurate diagnosis assay is essential to guide the clinical treatment and prognosis.

With unprecedented performance of high multiplexity, sensitivity and specificity, and throughput in a wide range of applications, mass spectrometry (MS) is one of emerging technologies in clinical diagnostics [5]. The US Food and Drug Administration (FDA) has approved a number of MS-based in vitro diagnostic methods, such as newborn screening tests for amino acids, vitamin D assay, free carnitine, and acylcarnitines using tandem mass spectrometry [6]. However, MS-based diagnostic devices that measure protein biomarkers are underutilized in clinical practice [7]. There is a limited number of FDA-approved diagnosis assays, such as MALDI-TOF MS for molecular microbiology diagnostics (e.g. VITEK® MS PRIME, MALDI Biotyper®); nearly all protein MS assays are laboratory developed tests (LDTs). In 1988, the United States congress amended the Public Health Services Act revising the federal program for certification and oversight of clinical laboratory testing.Under the current regulatory landscape, MS-based LDTs are subjected to oversight under the Clinical Laboratory Improvement Amendments (CLIA). It’s noteworthy that the FDA has expressed interest in extending its regulatory authority to cover LDTs in the future.

On the operation aspects, clinical labs have to meet stricter regulations to fulfill patient testing requirements compared to the research labs. Additional requirements in automation, standardized workflow, stringent criteria in assay performance, comprehensive documentation, staff training and competence, and quality management are critically needed to ensure robustness, reproducibility and accuracy for clinical testing [8]. Many research labs are engaged in translational research aimed at developing diagnostic assays. It is crucial to comprehend the practical aspects and additional requirements for clinical laboratories to successfully transition research assays into a clinical setting, ultimately benefiting patients directly.

Accompanying the increasing success of MS-based proteomics for large-scale protein characterization and biomarker discovery, there is an increasing demand to implement research protein MS assays to meet the requirements of clinical laboratories for their transition from bench to bedside. Among the MS assays, the targeted multiple reaction monitoring (MRM) MS has been widely used for the small molecule characterization under the current regulatory landscape. As a model demonstration, this project will use an immuno-MRM mass spectrometry-based assay for Tg quantification measurement to establish a clinical laboratory in Taiwan, meeting the CLIA guideline (termed as CLIA-equivalent laboratory) as compared to similar assays performed in the United States. Starting from the infrastructure of a research laboratory, we share the considerations, planning, preparation and the process to establish a clinical mass spectrometry assay in a research laboratory under CLIA requirements. The practical features beyond the considerations of a research lab, including laboratory facility, testing environment, instrument validation, assay development and validation, quality system, sample testing, and personnel competency, are discussed. Finally, proficiency testing was performed to confirm harmonization or standardization of the established Tg-iMRM MS assay between our laboratory and the University of Washington Medical Center, USA.

## Model assay: immuno-MRM thyroglobulin assay

The thyroglobulin (Tg) is a well-established serum biomarker used in the clinical management of patients after they have undergone treatment for thyroid carcinoma. Clinical laboratories measure the concentration of Tg in serum and plasma to detect signs of disease persistence or recurrence following initial treatment involving surgery and radioiodine ablation. However, the effectiveness of the commonly used immunoassay in human serum and plasma is constrained, as reported in a 2013 review [9]. This limitation arises from the presence of naturally occurring anti-thyroglobulin autoantibodies (TgAb) in a substantial portion of the population (10–30%), which can interfere with immunoassays, leading to erroneous measurement of underestimated Tg concentrations than the actual levels, especially in cases of differentiated thyroid carcinoma (DTC) recurrence. As a result, patients with positive TgAb levels often undergo additional radioiodine imaging scans to identify potential areas of persistent or recurrent disease [10]. Consequently, the clinical utility of the Tg biomarker serves as an example of how conventional immunoassay-based methods can be hindered by inherent limitations from patients.

To address the challenge presented by autoantibody interference in immunoassays, Hoofnagle and colleagues introduced an innovative approach: a quantitative immuno-multiple reaction monitoring (iMRM) mass spectrometry-based assay for thyroglobulin (Tg). This method was designed to bypass the issues caused by autoantibodies, as described in their study [11, 12]. In this assay, tryptic peptides derived from thyroglobulin are selectively captured using peptide-specific antibodies. Subsequently, these peptides are quantified via liquid chromatography-multiple reaction monitoring mass spectrometry (LC-MRM MS) with the assistance of external calibrators. These peptides serve as a proxy for quantifying the thyroglobulin protein in serum. Notably, this iMRM MS-based assay is now routinely employed at several reputable healthcare institutions, including the University of Washington Medical Center, Mayo Clinic, ARUP Laboratories, and Laboratory Corporation of America® Holdings (LabCorp).

## Planning for preparation, assay development, instrumentation, training, and testing

Compared to the current standard technologies in clinical diagnosis, MS-based technologies are far more complex in both instrumentation and data analysis, whose performance is influenced by many factors in sample matrix and handling, stability of infrastructure and technician skills. The establishment of a clinical laboratory involves a coordinated effort encompassing various aspects such as space allocation, workflow design, equipment selection, ventilation, lighting, plumbing, electrical systems, and data management. It is important to note that mass spectrometers are not always classified as in vitro diagnostic (IVD) instruments.Furthermore, the choice of method and mass spectrometry not only depends on the expense and maintenance of instrumentation but also requires thorough considerations for the return on investment. The evaluation list to establish a mass spectrometer is detailed in Table 1.

**Table 1:** Evaluation list to set up LC-MS/MS in a clinical laboratory

**Main Space**	
Mass Spec, HPLC, Computer, Liquid N2, Noise, and venting	
**Related Infrastructures**	
Bench: fixed |removable (recommended for easy access to the back for PM or services)	
Electrical system: dedicated electricity supply | UPS (NO UPS for GS-MS!)	
Venting System: organic solution used from MS	
Liquid nitrogen: N2 tanks (mostly recommended by manufactures) | generators (dual or triple generator)	
Noise: a separate space | Insulation	
Temperature: constant	
**Staff**	
A good med tech can be trained to run MS, especially when no method development involved	
MS requires constant attention and more technically challenge than the current clinical lab assays	
Data needs to be reviewed individually	
May not be good to everyone	
Can be treated as a career ladder	
**Timelines**	
Planning, evaluation, purchase, installation, training, testing; – 9 months to 1 year	
**Training**	
On-site preferred (one to two weeks)	
Create the methods for your specific needs	
**Methods to use**	
Choose the method you will use for your assay	
For organic extraction, fume hood or ventilation is required	
For automated, cost of the automation will be considered	
**Visit on site**	
Find somebody you know/trust who is currently using the MS you are interested and get feedback from them	
Send samples to companies to test the MS system (demo chemists)	
**Interface between MS data to current clinical database**	
No interface available from companies currently	
May need to hire a programmer to write an interface to transfer data (cost) to avoid human error from manual data transfer	
**Cost of the instrument**	
Instrument can be expensive	
Typically capital expenditure is required.	
Return on investment is determined by multiple factors	
**Cost of service contract**	
Suggest pre-buy if possible to get the current price and also with discounts	

**Fig. 1:**
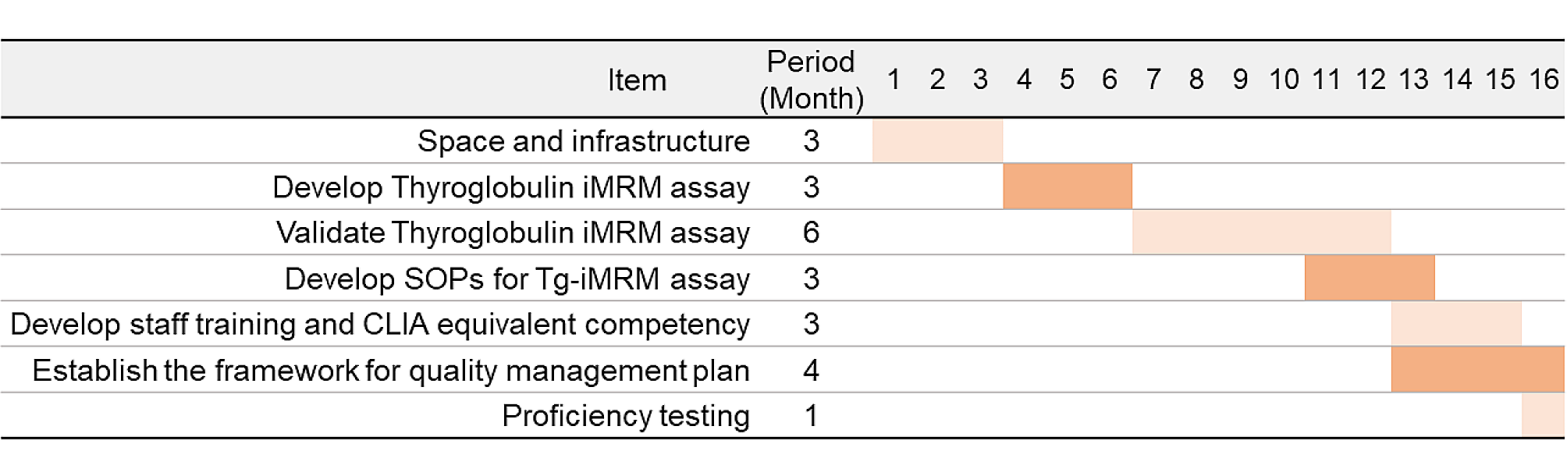
Preparation, timeline and milestone to establish an immuno-multiple reaction monitoring (iMRM) mass spectrometry-based thyroglobulin assay meeting the Clinical Laboratory Improvement Amendments (CLIA) guideline

When implementing technically complex mass spectrometry (MS) or liquid chromatography-mass spectrometry (LC-MS) procedures, a well-thought-out plan is essential. This process should align with Clinical Laboratory Improvement Amendments (CLIA) compliance, as depicted in Fig. 1, which provides a structured list of major milestones and expected outcomes for transitioning a research laboratory into a clinical laboratory. The initial step involves preparing the laboratory space and infrastructure for conducting assays. To successfully transfer and implement a fully developed MS assay at a different location, the development and validation of the assay, along with the establishment of standard operating procedures (SOPs), may require several months to a year to ensure comprehensive analytical accuracy.

***Staff training and competency*** Ensuring accurate, reliable, and timely testing is the next phase, which includes staff training and competency assessment adhering to CLIA standards. Compared to the operation of a research laboratory, this is a unique step to be part of quality management In a clinical laboratory, all the laboratory personnel training and competence assessment need to be planned, documented and evaluated. On a biannual or annual basis, staff competency needs to be evaluated and documented to demonstrate the necessary knowledge, skill and behaviors to perform their respective duties. Given the complexity of MS technology, proficient LC-MS/MS technical specialists are crucial. While certified laboratory technicians with a general clinical laboratory background can handle routine tasks and result reporting, mass spectrometry technical specialists are often needed for troubleshooting related to LC-MS/MS instrumentation. In this study, two certified staff members were trained for tasks like sample preparation, instrumentation operation, data analysis, and report generation. Service contracts were also acquired from the vendor for LC-MS/MS instrument troubleshooting and maintenance.

***Quality control*** The implementation of a quality management plan is a unique procedure that is not common practice in a research environment. It is crucial to establish guidelines and frameworks for maintaining quality throughout the project. Finally, proficiency testing is conducted through interlaboratory comparison testing to assess individual laboratory performance, ensuring competence and quality assurance. For the thyroglobulin (Tg) assay developed in this study, proficiency testing involved comparing quality control (QC) and clinical samples with the Clinical Chemistry Laboratory overseen by Dr. Andrew Hoofnagle at the University of Washington Medical Center in the USA.

### Planning for Laboratory infrastructure (Fig. 2)

**Fig. 2:**
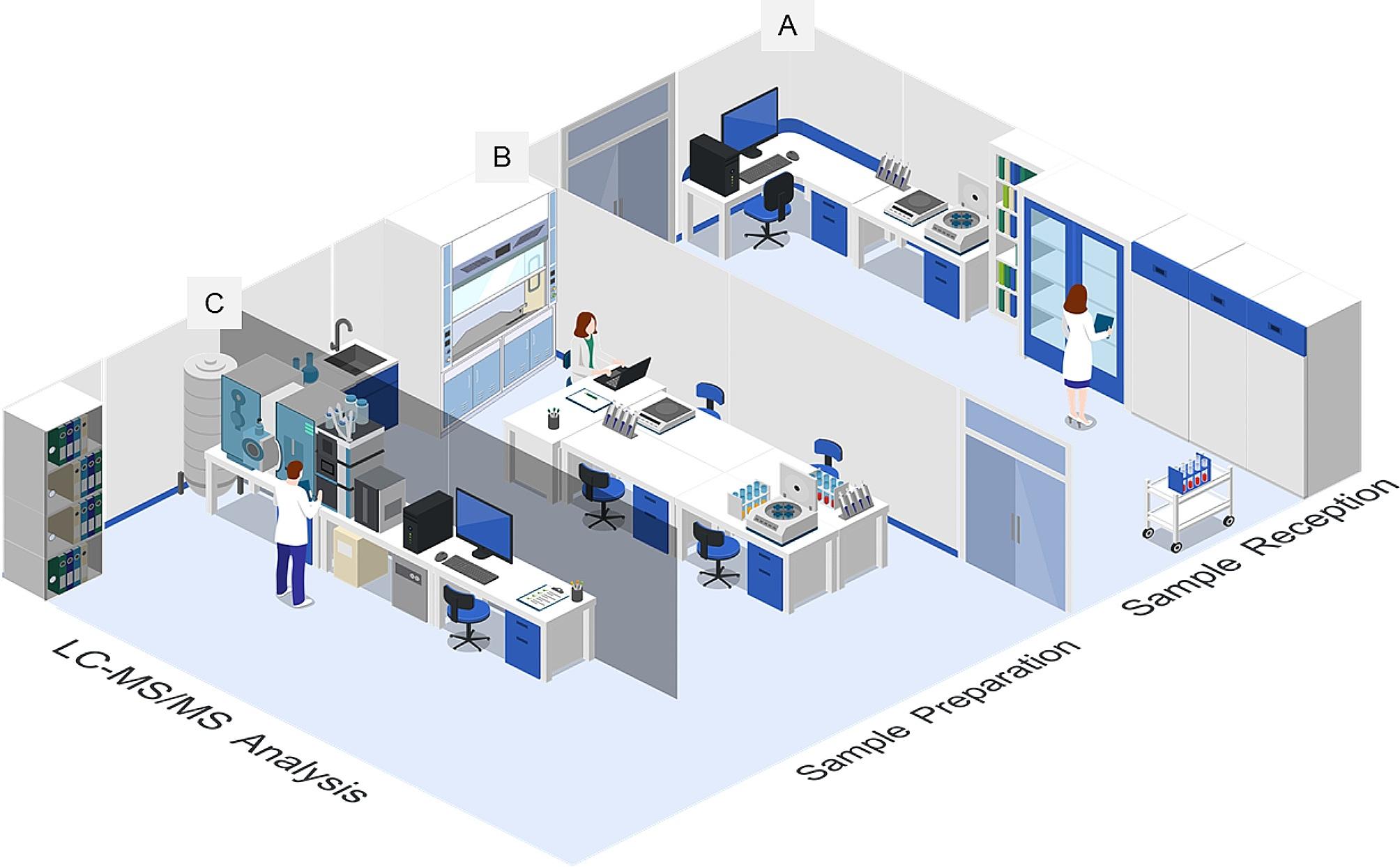
Laboratory layout for sample reception room **(A)**, sample preparation station **(B)**, and mass spectrometry analysis room **(C)**

While targeted protein mass spectrometry assays have gained significant popularity in biomarker discovery and validation, additional prerequisites must be met to make them practically applicable within the constraints of medical laboratory infrastructure. These prerequisites encompass various factors that can be controlled, including the laboratory environment, technician proficiency, instrument calibration, storage conditions, reagent and sample handling, and the quality of assay reagents (as cited in [13]). In contrast to research laboratories, where the primary focus often revolves around advancing technology, in a medical laboratory context, the emphasis is on maintaining strict consistency within the workspace and hardware instrumentation. This consistency is essential to ensure the robustness of operation, the quality of data generation, and adherence to stringent standards.

To transform a traditional biomedical research laboratory, a significant effort was made to plan and fully renovate the laboratory space. This newly designed space was carefully arranged to accommodate three main areas: the sample reception room, the sample preparation station, and the mass spectrometry analysis room, as depicted in Fig. 2.

***Sample reception room*** (Fig. 2A): This area serves as the initial point of entry for samples. It includes a designated space for inspecting incoming sample packages and containers to check for any damage. Additionally, the quality of the samples is assessed against established recruitment criteria. Following this, samples are registered, labeled with barcodes for proper tracking, and safely stored. The room layout allows for potential expansion to handle increased sample throughput if required.

***Sample preparation station*** (Fig. 2B): The sample preparation station is equipped with essential laboratory equipment, including a fume hood, clinical centrifuge, pipettes, thermomixer, and more. It is specifically designed for the transformation of thyroglobulin (Tg) proteins into peptides, which is a critical step in the analysis. The process involves several key steps. Initially, immunoaffinity (IA) beads are prepared by attaching Tg-peptide antibodies to magnetic beads. Next, serum samples undergo denaturation, alkylation, and trypsin digestion to produce peptides. These Tg peptides are subsequently enriched using the IA beads and are stored at a temperature of 4℃ in preparation for later LC-MS/MS analysis.

***Mass spectrometry analysis room*** (Fig. 2C): This room is equipped to accommodate the necessary components for mass spectrometry analysis. It houses the high-performance liquid chromatography (HPLC) equipment, the mass spectrometer, a computer for data acquisition and analysis, as well as auxiliary components such as a Liquid nitrogen tank, a nitrogen generator, a noise reduction device, and a waste venting line. The design and setup of this room follow the guidelines outlined in the “Site Planning Guide” provided by the mass spectrometer manufacturer. While this guide offers valuable information about minimum space requirements, electrical power needs, gas supplies, ventilation, computer connectivity, and operational conditions, it may not cover all the specific operational requirements for running a clinical mass spectrometry laboratory. Nonetheless, it serves as a fundamental reference point for the planning and setupprocess.

### Case study: building clinical laboratory infrastructure in Taiwan under CLIA requirement

**Fig. 3:**
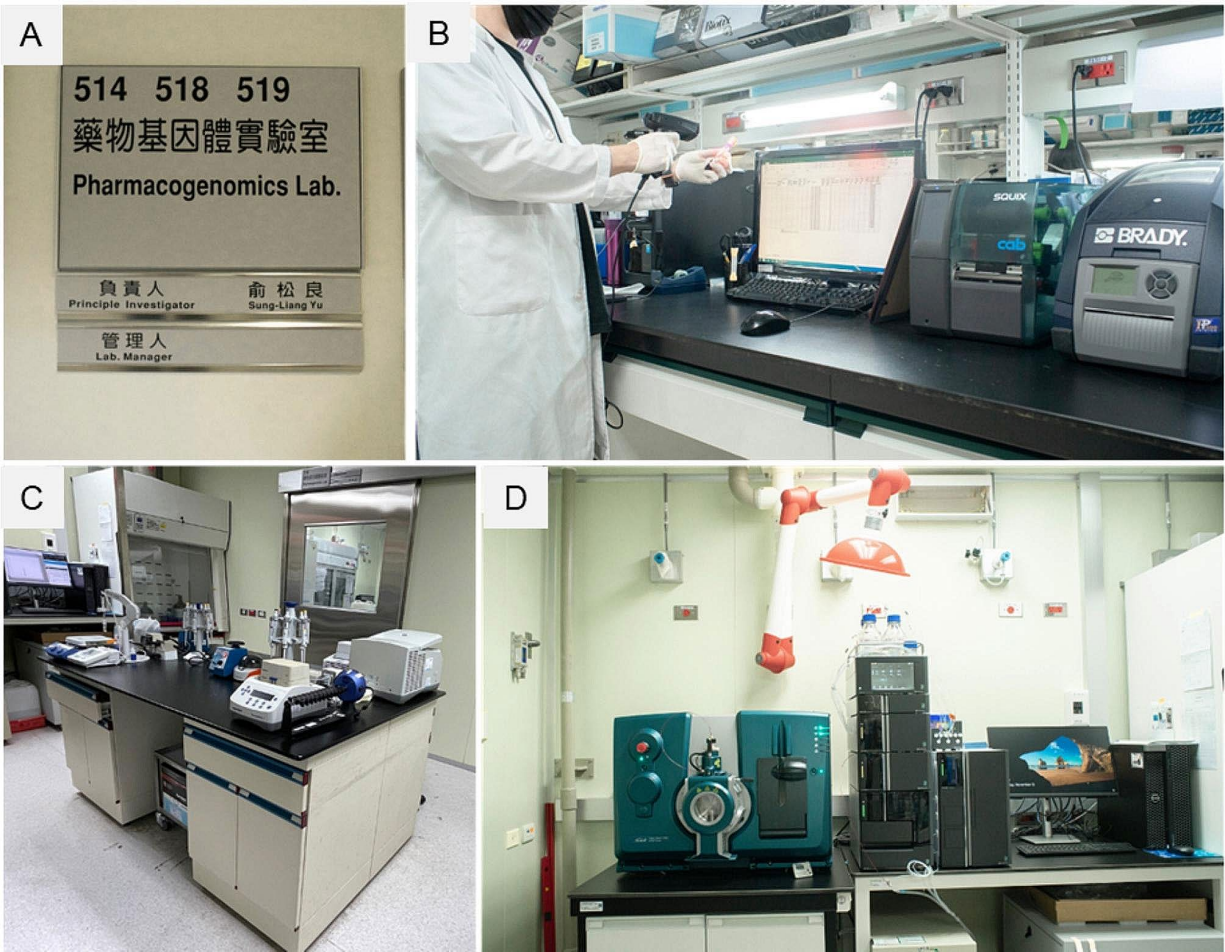
The CLIA-equivalent laboratory was established within the pharmacogenomics laboratory under the Centers of Genomic and Precision Medicine, National Taiwan University **(A)**. The laboratory comprised three distinct areas: **(B)** the sample reception room for barcode labeling and scanning and sample storage; **(C)** the sample preparation station for the transformation of Tg proteins into peptides; and **(D)** the mass spectrometry analysis room

As per the outlined plan, we started to establish the CLIA-equivalent laboratory, i.e. to convert a research laboratory to operate the Tg-MS assay under CLIA requirement, in the research space of the pharmacogenomics laboratory under the Centers of Genomic and Precision Medicine, National Taiwan University. This laboratory holds certification from the Taiwan FDA as a medical laboratory (equivalent to ISO15189 certification) for gene testing (Fig. 3). Given that the Tg-MS assay is intended for clinical diagnostics, it is imperative to validate the method’s quality and competence. In Taiwan, adherence to specific requirements for quality management in clinical assays is guided by the Laboratory Developed Tests and Services (LDTS) guidelines put forth by the Taiwan Food and Drug Administration (TFDA) and biomedical molecular testing method validation guidelines (TAF-CN-G38) monitored by Taiwan Accreditation Foundation (TAF), both literally following the ISO 15,189 international standards. For the assay development and validation, we followed the guideline outlined in the quantitative measurement of proteins and peptides by mass spectrometry (CLSI-C64) the Clinical & Laboratory Standards Institute (CLSI) guideline, a framework initiated by CPTAC and collaboratively developed by the research and clinical laboratory community, including academia, regulatory bodies, and industry, and published by the Clinical & Laboratory Standards Institute. At our site, we meticulously assessed the analytical merits of the Tg-MS assay, encompassing accuracy, precision, reportable range, cut-off value, specificity, and stability of the Tg-MS assay. The summary and comparison of regulatory requirements between the TFDA-LDTS and TAF-CNLA-G38 guidelines are presented in Table 2. Additionally, specific chapters in CLSI C64 were adopted for evaluating assay development and validation, and these are enumerated for comparative purposes.

**Table 2:** Comparison of regulatory requirement among CLSI, LDTS, and TAF for analytical performance in developing a LC-MS assay. TFDA-LDTS: Taiwan Food and Drug Administration; TAF: Taiwan Accreditation Foundation; CLSI: Clinical & Laboratory Standards Institute

	Item	TFDA-LDTS	TAF-CNLA-G38	CLSI-C64Ed1E
I	Tracebility	⌾		
II	Accuracy	⌾	⌾	8.10
	Trueness (bias)		⌾	8.10
	Method comparison			8.10
	Patient comparison			
III	Precision	⌾	⌾	8.2
	Reproducibility	⌾	⌾	8.2
	Repeatability		⌾	
	Measurement uncertainty		⌾	
IV	Reportable range	⌾		
	Linearity interval		⌾	8.4.1
	Measuting interval		⌾	
	Dilution verification			8.4.2
V	Sensitivity	⌾	⌾	8.3
	Cut-off value	⌾		8.12
	Limit of detection (LOD)		⌾	
	Limit of quantitation (LOQ)		⌾	
	Carry over			8.8
VI	Specificity	⌾	⌾	8.7
	Interfering substances	⌾		8.7
	Matrix effects		⌾	8.7
	Matrix comparison			8.11
VII	Stability	⌾		8.5/8.6
	Ion transition ratios			8.9
	Ruggedness		⌾	8.13.1
	Reagent lot changes			8.13.2

In the initial phase of setting up the laboratory facility, the primary tasks involve ensuring a dedicated electricity supply and the installation of a safe venting system for the disposal of waste generated from the use of organic solutions in the mass spectrometry (MS) process. As illustrated in Fig. 3, this photo depicts the CLIA-equivalent laboratory established within the Centers for Genomics and Precision Medicine at the National Taiwan University.

The repeatability and reproducibility of an assay depend significantly on the condition of the laboratory infrastructure. To ensure the generation of trustworthy and traceable measurement data, certain critical requirements must be met. These include a consistent and reliable supply of electricity, preferably backed up with an uninterruptible power supply (UPS), and well-controlled environmental conditions in terms of temperature and humidity within an air-conditioned room. These conditions should be both moderate and uniform.

Converting a conventional biochemical laboratory into a dedicated mass spectrometry analysis room requires a series of crucial modifications. Specifically, new electrical outlets, designed with specifications of 220 volts and 45-amp circuits, were installed. Additionally, a fume hood equipped with acid-base cabinets was incorporated into the lab. The inclusion of this fume hood necessitated the installation of new ducts to efficiently accommodate it and facilitate the proper ventilation of the mass spectrometry instrument. This process entailed various steps, including applying for departmental approvals and ensuring compliance with safety and regulatory requirements for power distribution. It’s worth noting that this entire transformation, from the approval stage to the completion of the renovation, could span several months due to the comprehensive nature of the modifications and the need for meticulous compliance with established standards and regulations.

Maintaining the appropriate temperature for mass spectrometry required the installation of an air conditioner with precise temperature control, along with an air compressor located in a separate room. To meet the nitrogen gas requirements of LC/MS applications, a nitrogen generator was put in place. To minimize noise within the lab, a well-placed nitrogen generator bench and the separation of the air compressor were practical solutions.

In summary, it is highly advisable to plan well in advance for such a lengthy and intricate process of transforming a conventional research laboratory into a CLIA-equivalent laboratory infrastructure. Moreover, it is crucial to recognize that, in contrast to a standard research laboratory, securing service contracts for equipment maintenance is paramount. These contracts encompass not only technological support but also the capability to troubleshoot issues, ensuring the sustained reliability of LC-MS/MS instruments over the long term.

### Assay development pipeline and demonstration of laboratory proficiency

Following the regulation and guidelines of LDTS and CLSI C64, we developed and validated the Tg-MS assay from the latest version established by Hoofnagle et al. [11]. in the medical laboratory at National Taiwan University, Taiwan. In brief, the unique peptide of Tg, FSPDDSAGASALLR (abbreviated as FSP), and its two dominant fragment ions (y7 and y12) are selected as target transitions for LC-MRM analysis to ensure the specific detection of Tg protein. Pairs of synthetic internal standards (SIS) of light and heavy isotope-labeling (IS) of the same sequence of FSP peptides are prepared to optimize the LC-MS/MS and validate the retention time of the FSP peptide in the LC-MRM. Further optimizations on the instrument settings such as collision energy and LC gradient are required for enhancing the transition signals of both target and SIS peptides. In our case, the two transitions have different optimal collision energies; 33.2% and 38.2% for y7 and y12 transitions, respectively. Compared to the common practice for a research assay, the additional key steps for a clinical assay development and validation of performance meeting regulatory guideline are summarized as follows:

***Quality control standards*** To develop the Tg-MS assay, we adopted a commercially available human serum as Tg standard to prepare calibrators and quality control samples (QCs). The initial concentration of Tg in the commercial human serum was determined as 13.12 ng/mL by LC-MRM assay from the certified clinical laboratory in the UW Medical Center. With this human serum, we prepare 5 calibrators with Tg concentrations ranging from 0.23 to 13.12 ng/mL. Three QCs of different Tg concentration, including QC-high (10.99 ng/mL), QC-Mid (3.3 ng/mL) and QC-low (0.99 ng/mL), are designed for evaluation of the assay quality. It is recommended to prepare a large batch of calibrators and QCs to support at least half- or one-year use to ensure the consistency and stability of the assay.

***Quantification performance*** For every batch of Tg-MS assay, 400 µL of 5 calibrators, 3 QCs and a number of clinical samples (with IRB approval and informed consent) are aliquoted to undergo denaturation, reduction, and alkylation processes, following by the addition of SIS peptide, tryptic digestion, and the affinity purification of FSP and SIS peptides using peptide immunoaffinity enrichment with a well-characterized monoclonal antibody against FSP peptide. All the samples are then analyzed by LC-MRM. Then, we construct the calibration curve by using these 5 calibrators. A linearity with r^2^ higher than 0.95 in the calibration curve is accepted for measuring the Tg levels in three QCs and other clinical samples. For acceptance of the assay performance, the measured Tg levels of three QCs should be less than 20% deviation from the expected values. Under these two conditions, the measurement of Tg in the clinical samples demonstrated the quality of LC-MRM assay and the reliability of the results.

***Evaluation of analytical merits*** In order to validate the performance of Tg-MS assay at our site, we performed a series of measurements of QCs to evaluate the analytical merits that adhere to the LDTS and CLSI C64 guideline, including reportable range, accuracy, precision, sensitivity, specificity, interference, and stability of the assay (Table 3). Specifically, we performed 4 replicate analyses of 2–3 QCs per day for at least 3 —5 days to evaluate the within-day repeatability as well as 1 replicate analysis of 2–3 QCs for at least 7–10 days to evaluate the between-day reproducibility. Our data shows that the coefficient of variation (CV) of all three QCs in within-day and between-day experiments were 5 – 10% and < 10% CV, respectively. Furthermore, collecting a long-term result of QCs showed that the deviation between the measured and expected values of Tg in QC-mid and QC-high are 7.8% and 8%, respectively. These results demonstrated a precise and accurate measurement of serum Tg levels by the Tg-MS assay at our site. We also evaluated the interference from hemolysis by mixing up one hemolyzed serum (250–500 mg/dL) and non-hemolyzed serum in specific ratios to prepare 0%, 25%, 50%, 75%, and 100% hemolyzed serum samples. The data indicated that a serum sample with up to 25% hemolysis (62.5–125 mg/dL) is tolerated with Tg-MS assay.

**Table 3:** The overview of analytical performance of Tg-iMRM-MS established in Taiwan

Item		Details	Result	
**1. Assay development**				
Transition selection		Target and internal standard peptides	FSP2 (704.04/586.90, 704.04/687.5), FSP4 (709.04/591.89, 709/04/697.5)	
Collision energy optimization		Default ± 10	y7: 33.2; y12: 38.2	
**2. Reportable range**				
Calibration curve		Standard serum or pooled clinical serum, serial dilution	0.2 ~ 13 ng/mL	
LOD		Lowest detectable concentration	0.1 ng/mL	
LOQ		Lowest quantifiable concentration, CV < 20%	0.2 ng/mL	
**3. Accuracy**		Evaluated by QC samples, deviation < 20%	QC_mid: 7.8%; QC_high: 8%	
**4. Precision**		Evaluated by QC samples, deviation < 20%		
Within-day repeatability		2 or 3 QC samples, 4 replicates/day, 3 ~ 5 days	QC_low: 10.07%; QC_mid: 7.52%; QC_high: 5.49%	
Between-day reproducibility		2 or 3 QC samples, 1 replicate/day, 7 ~ 10 days	QC_low: 9.82%; QC_mid: 7.97%; QC_high: 9.92%	
**5. Sensitivity**		LOQ	0.2 ng/mL	
**6. Specificity**		Signal overlay of endogenous and internal standard peptides		
**7. Interference**		Hemolysis, deviation < 20%	< 25% hemolysis (62.5–125 mg/dL) is tolerated	
**8. Stability**		Room temperature (20 °C); 4 °C; -80 °C	RT: 48 h; 4 °C: 48 h; -80 °C: > 1yr	
**9. Proficiency test**		20–35 QCs and clinical samples exchanged with reference laboratory	r^2^ = 0.996, slope = 0.9287	

***Proficiency test*** Finally, proficiency testing was performed to confirm harmonization or standardization of the established Tg-iMRM MS assay between our laboratory and the University of Washington Medical Center. Proficiency testing (PT) is a very important concept for clinical assays which is very foreign to research laboratories. PT is a long-term evaluation of the accuracy and reliability of the assay. CLIA requires proficiency testing for all assays in a clinical laboratory on a routine basis and these results will be graded and evaluated. Consecutive failures of PT results for an assay will result in discontinuation of the assay in the lab. The PT can be part of a program such as CAP or self-arranged with other laboratories. In our particular case, there is no official CAP program for Tg PT. We established a PT process with the Washington University Medical Center.

In our protocol, only the Tg antibody and affinity bead were the same with the University of Washington Medical Center, most of the chemicals and reagents as well as the LC-MS system were different. Thus, proficiency testing with a routine exchange of QC and clinical samples between multiple clinical laboratories is necessary to provide reliable results using distinct vendors, models, platforms or institutions [14]. As shown in Table 3, we exchanged 35 QCs, clinical samples, and diluted clinical samples with UW Medical Center to compare the measured Tg levels between two sites. The Tg measurements between two sites are linear and highly correlated (r^2^ = 0.996 and slope = 0.9287). The concordant results across a small set of patients with and without TgAb demonstrated the standardization between the two laboratories and quality assurance of the established CLIA-equivalent Tg-iMRM MS assay in Taiwan.

## Conclusion and perspectives

In summary, we have successfully established the first Tg-iMRM MS assay based on CLIA requirements in Taiwan. Notably, none of the previously published Tg-MS papers have conducted study on the Asian patient and their corresponding outcomes. Given that the Taiwanese population is representative of the broader East Asian cohort, this assay will undergo evaluation in a prospectively collected cohort. The findings from this assessment will play a crucial role in gauging the efficacy of the assay for clinicians and patients in the Asian context. The insights gained from this experience are poised to inspire and guide the future development of innovative protein MS assays.

Mass spectrometry is a promising clinical assay of choice with its inherent multiple strengths in characterization of thousands of proteins, multiplexing capacity, remarkable sensitivity and specificity, and compatibility with various types of specimens, which is often not achieved by other analytical techniques. Despite these advantages, challenges such as instrument complexity, cost, and the need for specialized expertise in MS technology exist. Although the MS-based assays are still underutilized in the current clinical environment, the active research in proteomics biomarker discovery have inspired rapid technological advancement and increasing implementation of mass spectrometry assays as a method of choice in clinical laboratories. As the field continues to evolve, mass spectrometry-based diagnosis assays are likely to have increasing integration into the clinical pipeline for precision medicine and improving patient care.

### Experimental session

#### Serum collection from patient with thyroid cancer

Patients with resectable thyroid cancer who consent to donate their follow-up serum samples were recruited. The study was approved by the Institutional Research Board of the National Taiwan University Hospital, Taiwan.

#### Immunoaffinity purification of tg peptide

To prepare the anti-Tg beads, briefly, the tosyl activated beads (company) were mixed with 100 mM sodium borate, and then conjugated with Tg antibody (Abcam) at 37 °C overnight, followed by blocking with PBS/0.1% BSA and Tris/0.1% BSA, and then stored in PBS/0.1% CHAPS /0.02% NaN3 before use.

400 µL of serum from calibrator, QCs, and clinical samples were aliquoted for reduction with 20% DOC /0.19 M Tris/0.03 M TCEP for 60 min at 40 °C and alkylation with 0.06 M IAA for 30 min at 37 °C in the dark. One hundred µL of 10 mg/mL Trypsin in 10 mM HCl was added into each sample for 40 min at 37 °C and then 20 µL of 3 mg/mL TLCK (trypsin inhibitor) in 10 mM HCl was added to quench the digestion.

The anti-Tg beads were added to the tryptic digest for 60 min at room temperature. After removing the supernatant through magnet, the anti-Tg beads were washed with PBS/0.1% CHAPS for three times and then the captured Tg peptides were eluted from the beads by using 2.5% acetic acid.

#### LC-MRM analysis

The purified Tg peptides from calibrators, QCs and clinical samples were reconstituted in 2.5% acetic acids and subjected to LC-MRM analysis using the 1290 Infinity II LC System (Agilent, xx) coupled with QTRAP® 5500 LC-MS/MS (Sciex, AB SCIEX Concord, ON, Canada). Peptides were injected into Acquity UPLC HSS T3 column (1.8 μm pore size, 2.1 × 50 mm) and elutedusing a gradient composed of mobile phase comprising (A) 0.1% formic acid in water and (B) 0.1% formic acid in methanol over a 7-minute period. The gradient of buffer (B) transitioned from 20 to 67% in 1 min at a flow rate of 0.3 mL/min, followed by an increase from 67 to 95% in 1 min at a flow rate of 0.6 mL/min. Subsequently, it returned to 20% in 1 min at a flow rate of 0.6 mL/min, maintaining this composition for 1 min at a flow rate of 0.3 mL/min to re-establish column equilibrium.

The mass spectrometer was operated in positive mode, employing the following parameters: an ion spray voltage (IS) of 5500 V, a curtain gas (CUR) pressure of 30 psi, a temperature (TEM) of 550 °C, and ion source gasses 1/2 (GS1 and GS2) set at 50 psi. Analyte-dependent parameters included maintaining the declustering potential (DP) at 125 V, entrance potential (EP) at 10 V, and collision cell exit potential (CXP) at 10 V. For the targeted Tg peptide FSP, unit resolution for both Q1 and Q3 quadrupoles was applied to select the precursor ions. The QTRAP 5500 mass spectrometer was operated with optimized collision energy (CE) and declustering potential (DP) for the two transitions: the y12^++^ ions and the y7^+^, with collision energies (CE) optimized at 33 and 42 V, respectively.

#### Data processing and Tg quantitation

The selection of all transitions for each peptide and data processing were performed using the Skyline software [15]. The MRM MS raw files were processed using Skyline (version 22.2.0.527), where transitions were matched against the Tg target peptide library sequence. Two transitions of the targeted peptide were chosen, covering y7^+^ and y12^++^ ions for FSP. Retention time was configured for automatic peak detection and integration, with manual adjustments made as necessary for integration limits. Using Skyline, two transitions of high intensity and same retention time within ± 6 s compared to the heavy peptide were selected. The Skyline automatically calculated the peak area for both light (synthetic or endogenous) and corresponding heavy IS peptides. Subsequently, the peak area ratios of light and heavy peptides (light-to-heavy ratio) were computed for establishing calibration curves and quantifying peptide abundance.

To determine the endogenous Tg concentration in patients, a quantitation curve was constructed by serial dilution of Tg targeted peptide (light isotope) of known concentration and spiking its heavy isotope-labeled counterpart at a fixed concentration (20 pmol). To generate Tg targeted peptide for the calibration curves, human serum was mixed and serially diluted with rabbit serum at five concentrations. Fixed amounts of heavy peptides (50 nmol/L) were consistently maintained as internal standard and spiked into the sample. For absolute quantification of the Tg using the targeted peptide, the light-to-heavy peak area ratios of the two transitions were averaged. The ratios of the calibrators were inputted into their regression equation against concentration through linear regression analysis. For quantification, the light to heavy ratio of the quality control or clinical samples are obtained. The resulting ratios from the endogenous Tg were then input into the regression equation to calculate its concentration (ng/mL).

## Data Availability

No datasets were generated or analysed during the current study.
